# Comparative assessment of the biological activity of the green synthesized silver nanoparticles and aqueous leaf extract of *Perilla frutescens* (L.)

**DOI:** 10.1038/s41598-023-33625-x

**Published:** 2023-04-19

**Authors:** Mansoureh Tavan, Parichehr Hanachi, Mohammad Hossein Mirjalili, Abolfazl Dashtbani-Roozbehani

**Affiliations:** 1 Department of Biotechnology, Faculty of Biological Science, Alzahra University, Tehran, Iran; 2 Department of Agriculture, Medicinal Plants and Drugs Research Institute, Shahid Beheshti University, Tehran, 1983969411 Iran; 3 College of Science and Engineering, Flinders University, Bedford Park, SA 5042 Australia

**Keywords:** Biochemistry, Biotechnology, Cancer, Microbiology, Plant sciences, Nanoscience and technology

## Abstract

Green synthesized nanoparticles (GSNPs) display fascinating properties compared to physical and chemical synthesized ones. GSNPs are currently used in numerous applications such as food packaging, surface coating agents, environmental remediation, antimicrobial, and medicine. In the present study, the aqueous leaf extract of *Perilla frutescens* L. having suitable capping, reducing, and stabilizing compounds was used for green synthesis of silver nanoparticles (Pf-AgNPs). The bioreductant capacity of aqueous leaf extract of *P. frutescens* for Pf-AgNPs was determined by different confirmatory techniques including UV–Visible spectroscopy, XRD, FESEM, EDX, zeta potential, DLS, SERS, and FTIR analysis. The results exhibited that Pf-AgNPs had optimal size (< 61 nm), shape (spherical), and stability (− 18.1 mV). The antioxidant activity of Pf-AgNPs with both DPPH and FRAP assays was significantly higher compared to *P. frutescens* extract. Furthermore, Pf-AgNPs had high antimicrobial activity against *Escherichia coli* and *Staphylococcus aureus* (MIC = 0.78 mg/mL), and *Candida albicans* (MIC = 8 mg/mL) while the plant extract showed low antimicrobial activity against both bacterial strains and the fungus tested. Pf-AgNPs and *P. frutescens* extract also exhibited moderate toxicity on MCF-7 cancer cells with IC_50_ values of 346.2 and 467.4 µg/mL, respectively. The results provide insights into using the biosynthesized Pf-AgNPs as an eco-friendly material for a wide range of biomedical applications.

## Introduction

In recent years, nanotechnology has attained great attention from different fields such as chemistry, biotechnology, medicine, applied microbiology, physics, and material sciences^1^. The three terms “creation”, “exploitation”, and “synthesis” are related to nanotechnology, which commonly investigates materials with a size of less than 1 mm^2^. There are two general methods for the synthesis of nanoparticles (NPs): top-down and bottom-up methods^3^. In the top-down method, NPs are produced by reducing the size of a proper starting material which is limited in use due to defects in the surface structure of the product. In bottom-up synthesis, NPs are made from smaller structures such as atoms and molecules using biological and chemical methods^4^. Bionanotechnology or green synthesis is a cost-effective and eco-friendly technique used for forming the NPs using microorganisms and plant extracts obtained from plant tissue, fruits, and marine algae^5^. Organic and inorganic NPs are currently utilized in various fields including biomedicine, electrochemistry, pharmaceuticals, cosmetics, and food technology due to their unique physical and chemical properties. Inorganic NPs include metallic NPs (Cu, Ag, Au, Pt, Pd, and Al), semi-conductor NPs (CdS, ZnS, and ZnO), and magnetic NPs (Ni, Fe, and Co), while carbon NPs including carbon nanotubes, quantum dots, and fullerenes are known as organic NPs^6^. In general, NPs possess very small size (< 100 nm) and higher surface area to volume ratio causing excellent catalytic and biological properties compared to bulk materials^7^. Silver NPs (AgNPs) have been extensively considered compared to other metal NPs due to their vast application in chemistry, pharmacology, microbiology, and food technology^8,9^.

Generally, there are several methods for the synthesis of AgNPs including chemical vapor deposition, sol–gel, thermal decomposition, hydrothermal microwave-assisted combustion etc^10^. In the early 1900s, green synthesis of AgNPs via biomaterials such as plant extract as reducing agents has been known^11^. The source of the plant extract with different concentrations and combinations is effective on the characteristics of NPs. Indeed, AgNPs can generate through the oxidation of Ag^+^ to Ag^0^ using various biomolecules including phenolics, terpenoids, alkaloids, and the proteins presenting in the extracts of diverse plant parts such as leaves, roots, fruits, and peels^12,13^. These AgNPs have extensive applications in biomedical fields including biosensors, biomolecular detection, and antibacterial, antifungal, and antiangiogenetic agents^14,15^. It has also been reported that silver as a safe antibacterial metal possesses the capacity of killing more than 650 pathogenic bacteria by destroying the bacterial cell membrane and cell lysing^16^. In addition, the efficiency of AgNPs toward cancer cell death via destroying the mitochondrial respiration chain in cancer cells and degrading DNA has been proved^17^. Green synthesis of AgNPs using the plant extracts such as *Eugenia roxburghii* DC.^18^, *Scutellaria multicaulis* Boiss.^19^, *Heracleum persicum* Desf.^20^, *Origanum vulgare* L.^21^, and *Salvia spinosa* L.^9^ as well as their biological applications have been previously reported.

*Perilla frutescens* (L.) Britt. is an annual edible plant belonging to the family Lamiaceae that is native to China, Japan, Korea, India, and Southeast Asia^22^. Also, *P. frutescens var. crispa* (green-purple leaves) is a valuable medicinal plant that grows as a wild plant in north of Iran^23^. Among diverse cultivars of *P. frutescens*, three are broadly used by local people which are generally known as *var. frutescens* (green leaves), *var. crispa* (green-purple leaves) and *var. acuta* (red–purple leaves). A high concentration of anthocyanins (especially cyanidin-type anthocyanin) lead to the diversity of leaf color^24^. This plant is mainly consumed as fresh vegetables, food ingredients, and medicinal herbs. The leaves of different varieties contain a wide range of bioactive compounds such as phenolic acids (rosmarinic acid, caffeic acid, ferulic acid, and vanillic acid), flavonoids (apigenin, catechin, luteolin, rutin, and scutellarein), coumarins (6.7-dihydroxycoumarin and esculetin), anthocyanins (malonyl shisonin, Cis-shisonin, and shisonin), carotenoids, sesquiterpenoids, neolignans, fatty acids and other constituents^25,26^. Amongst these compounds, phenolic acids and flavonoids are the most important bioactive compounds of the plant, which are responsible for the reduction of Ag ions to Ag NPs^27^. Moreover, the extracts of *P. frutescens* have been well known for their potential antioxidant, anticancer, antimicrobial, anti-depressant, and insecticidal activities due to the presence of bioactive compounds present in the plant as reported previously^28^.

Studies on green synthesized nanoparticles (GSNPs) including AgNPs using various plant extracts and their increased biological potentials make the topic interesting to be researched further using more medicinally significant plants to improve/add to their biological potential since various plants have divers phytochemical compositions^29^. Using this idea, the valuable medicinal properties and the exciting bioactive compounds of *P. frutescens* (which make it potentially more worthy for the synthesis of AgNPs) promoted us to green synthesis of silver nanoparticles using the aqueous leaf extract of *P. frutescens* (Pf-AgNPs).

Although the synthesis of AgNPs by aqueous leaf extract of *P. frutescens* has been recently reported^27^, but the growth conditions including climatic and environmental conditions, geographic origins, and genetic variation can cause many changes in the content of bioactive compounds of plants. There are also few studies on this native species in our country. Therefore, the present study was aimed to evaluate the content of important bioactive compounds of aqueous leaf extract of *P. frutescen* as a native species by HPLC analysis and then to synthesize Pf-AgNPs. Also, in this study, an attempt was made to assess and to compare the antioxidant, antibacterial, antifungal, and anticancer activity of Pf-AgNPs and aqueous leaf extract of *P. frutescen*. Hence, our present investigation will help to evaluate the biological effects of Pf-AgNPs, and thereby Pf-AgNPs may be considered in nutraceutical, pharmaceutical and biomedical industries.

## Materials and methods

### Plant material and preparation of plant extract

The plant aerial parts were collected with the permission of local authorities from Siahkal, Guilan, Iran (latitude 37.1528 and longitude 49.8708), at the pre-flowering stage. The plant was identified by an expert botanist, Prof. Ali Sonboli, and a voucher specimen (MPH-3815) has been deposited at the herbarium of Medicinal Plants and Drugs Research Institute, Shahid Beheshti University, Tehran, Iran. The collection of plant and experimental research on the plant used for the study complies with relevant institutional, national, and international guidelines and legislation. The plant leaves were washed and dried in the shade at an ambient temperature. Powdered plant leaves (5 g) were heated up in 100 mL of distilled water at 70 °C for 60 min. Then, the cooled and filtered solution was preserved at 4 °C for further use.

### Biosynthesis of AgNPs with *P. frutescens* leaf extract

For the green synthesis of Pf-AgNPs, AgNO3 in three concentrations of 0.5, 1, and 2 mM was dissolved in *P. frutescens* extract aqueous solution (1, 2.5, 5, and 10%, v/v), and the mixture was sonicated for 90 min at 60 °C and in the dark condition. After that, the solution was centrifuged at 9000 rpm for 30 min and at 25 °C. Then, the sediment obtained was washed three times and stored for determining characterization and biological studies. The whole reaction was carried out in the dark to prevent photo-activation^30^.

### Characterization of Pf-AgNPs

At first, absorption maxima at the range of 200–800 nm were recorded by UV–Visible spectrophotometry (Unico 2100) for indicating the formation of AgNPs. Crystalline Pf-AgNPs was evaluated by X-ray diffraction (XRD) analysis. Fine powdered Pf-AgNPs were drop coated on XRD grid to make a thin film, and then the spectra pattern were recorded by using a Rigaku-Ultima IV diffractometer in Cu-Kα radiation wavelength of λ = 0.154178 nm at room temperature in a 2*θ* range 7 to 80*θ* at a scan rate of 0.2°/min. with a time constant of 2 s in 2 h. The topographical, size, and morphological properties of Pf-AgNPs were characterized by FESEM-EDX (ZEISS Sigma 300, Germany). A small drop (0.1 mL) of aqueous solution of nanoparticles was placed on the glass slide to create a thin film and then air-dried and coated by sputtering with gold. Zeta potential and dynamic light scattering (DLS, VASCO Cordouan Technology, France) were utilized to determine the surface charge and hydrodynamic diameter of Pf-AgNPs, respectively. Fourier transform infrared (FTIR) analysis (Perkin Elmer, USA) was used for distinguishing functional groups on the surface of Pf-AgNPs. The lyophilized Pf-AgNPs powder were mixed with potassium bromide (KBr) crystal as a beam splitter (1 mg/100 mg) and studied for the presence of IR bands in the range of 400–4000 cm^−1^ at a resolution of 4 cm^−1^ in the transmittance mode. Surface-enhanced Raman spectroscopy (SERS) analysis was performed for Pf-AgNPs by TakRam N1-541 spectrometer (Iran) equipped with a Hamamatsu detector equipped with a maximum power of 100 mW was used to obtain SERS activity of Pf-AgNPs. All spectra were collected at 10 mW of power, 1 s. of integration time for six accumulations^20^.

### Analysis of phenolic compounds of *P. frutescens* aqueous leaf extract by HPLC

High performance liquid chromatographic (HPLC) analysis was performed using a Waters liquid chromatography apparatus (2695, USA) and a PDA Detector waters (996, USA) as described by Tavan et al.^31^. As mentioned in the section of preparation of the plant extracts, powdered plant leaves (5 g) were heated up in 100 mL of distilled water at 70 °C for 60 min. A gradient solvent system was employed. The mobile phase consists of three solvents: (A) 1% acetic acid in water, (B) acetonitrile and (C) methanol and the following gradient program was performed: at 0 min, the A:B:C proportion was 90:0:0; at 10 min, 80:4:16; at 25 min, 75:5:20; at 30 min, 65:5:30; at 31 min, 40:0:60; at 37 min, 35:20:45; and at 50 min, 20:80:0. The system was allowed to run for another 5 min at 100% B in order to clean the column before re-equilibrate it at the initial conditions. The phenolic compositions (ferulic acid, rutin, caffeic acid, and rosmarinic acid) were recognized by comparing their retention times with those of the standards. Also, content of components was determined using their standards calibration curve.

### Determination of total phenolic and total flavonoid contents

The total phenolic content (TPC) of Pf-AgNPs and *P. frutescens* leaf extract was examined through the Folin–Ciocalteu method^32^ and it was calculated as mg/mL gallic acid (mg GAE/mL) using a calibration curve (*y* = 0.0176 + 0.0046x; *r2* = 0.9914). The extract of plant and Pf-AgNPs (0.3 mL, individually) was mixed with 1.5 mL Folin reagent (diluted: 10 v/v). The mixture was kept for 5 min, followed by the addition of 1.2 mL Na_2_CO_3_ (7% w/v). After 30 min of incubation at room temperature and the dark, the absorbance was read at 760 nm using a spectrophotometer (Unico 2100).

Also, the total flavonoid content (TFC) was measured according to the assay as described previously^33^. The extract of plant and Pf-AgNPs (0.275 mL, individually), 0.825 mL of distilled water, and 0.3 mL of a 5% NaNO_2_ solution were mixed. After 6 min, 0.6 mL of a 10% AlCl_3_∙6H_2_O solution was added. The mixture was kept for 5 min, followed by the addition of 2 mL of 1 M NaOH. Finally, 1 mL of distilled water was added to the test tube and mixed well. The absorbance was read instantly against the blank at 510 nm. The TFC was calculated as mg/mL quercetin (mg QE/mL). The calibration equation for quercetin was *y* =  − 0.0055 + 0.0063*x*; (*r*2 = 0.9976).

### Antioxidant activity

2,2-Diphenyl-1-picrylhydrazyl (DPPH) and ferric reducing/antioxidant power (FRAP) assays were selected for determining the antioxidant capacity of the Pf-AgNPs and *P. frutescens* leaf extract with concentrations of 0.1–1 mg/mL. The DPPH assay was performed according to the method of Huang et al.^34^. Each of extract of plant and Pf-AgNPs (0.075 mL) was reacted with 2.925 mL of 60 μM DPPH. The reaction mixtures were well shaken and incubated in the dark for 30 min after which absorbance readings were taken at 517 nm. The ascorbic acid was used as standard. The ability to scavenge DPPH radical was computed by the following equation:

$$\% {\text{ DPPH radical inhibition }} = \, \left[ {\left( {{\text{Abs}}_{{{\text{control}}}} {-}{\text{Abs}}_{{{\text{sample}}}} } \right)/{\text{Abs}}_{{{\text{control}}}} } \right] \, \times { 1}00.$$

The FRAP assay was estimated as described before^35^. The plant extract and Pf-AgNPs individually (0.2 mL) were added to 1.8 mL of FRAP reagent. The samples were placed at 37 °C for 5 min and then the absorbance was read at 593 nm. The FRAP values of the samples were computed from the standard curve equation and expressed as µg FeSO4/mg sample.

### Antimicrobial activity

Antimicrobial activities of Pf-AgNPs and *P. frutescens* leaf extract were estimated by the microdilution test against fungus strain of *C. albicans* (ATCC 10231) and bacteria strains including *E. coli* (ATCC 25922), and *S. aureus* (ATCC 25923)^36^. Minimum Inhibitory Concentration (MIC), Minimum Bactericidal Concentration (MBC) and Minimum Fungicidal Concentration (MFC) of samples were evaluated as described in our recent study^37^. The MIC of Pf-AgNPs and plant extract were evaluated for 3 microorganisms in triplicates by double fold serial microdilution test using 96-well microliter plates according to the Clinical laboratory standard Institute (CLSI). The plant extract and Pf-AgNPs were prepared (50 mg/mL) and serially diluted with Mueller–Hinton broth for bacterial (0.006–12.5 mg/mL) and fungal (0.031–32 mg/mL) cultures. The tested bacteria and fungus were grown in Mueller Hinton Broth (MHB, Hi-Media) for 18–24 h, followed by the matching of bacterial suspension to the turbidity equivalent to 0.5 McFarland solution (0.5–1 × 105 CFU/mL) with the addition of normal saline. The microplates were incubated for 24 h at 35 °C. The safexim (0.5–4 µg/mL, for *S. aureus* and *E. coli*) and nystatin (32–64 µg/mL, for *C. albicans*) were used as standard antibiotics. The first row of tube served as the negative control (200 µL Mueller–Hinton broth only). The MBCs and MFCs were detected by sub-cultivation of loop ful (0.001 mL) the test dilution from 96 well plates used to estimate MIC onto Mueller–Hinton broth plates. The plates were incubated at 35 °C overnight and the lowest concentration, without visible growth, was expressed as MBC or MFC, indicating 99.5% killing of the main inoculum.

### Biocompatibility and cytotoxicity analysis by MTT assay

Biocompatibility and cytotoxicity activity of Pf-AgNPs and leaf extract of *P. frutescens* with different concentrations of 600–37.5 µg/mL was considered by 3-(4, 5-dimethylthiazol-2-yl)-2, 5-diphenyltetrazolium Bromide (MTT) test against a normal fibroblast cell line (L-929) and a human breast cancer cell line (MCF-7) as described by Gomathi et al.^38^. Both cell lines were grown in RPMI-1640 medium (Gibco) with 10% FBS (Gibco) supplemented with antibiotics (100 U/mL penicillin and 100 µg/mL streptomycin). Cells were maintained at 37 °C under humidified air containing 5% CO2 and were passaged using trypsin/EDTA (Gibco) and phosphate-buffered saline (PBS) solution. After 24 h incubation, cells were treated with five concentrations (37.5, 75, 150, 300, and 600 µg/mL) of the suspension of Pf-AgNPs and aqueous leaf extract of *P. frutescen*. Untreated cells were used as controls. The drug cisplatin (75 µg/mL) was used as a standard. Cells were then rinsed with phosphate-buffered saline (PBS) and incubated with serum free medium containing 200 µL MTT for a further 4 h in dark at 37 °C. Subsequently, the supernatant was withdrawn, and the resulting crystals were dissolved with 100 µL of DMSO with gentle shaking at 37 °C for 5 min. Cell viability were quantified by measuring absorbance at 570 nm using an ELISA reader (Model wave xs2, BioTek, USA). The concentration of Pf-AgNPs and the extract of plant that resulted in 50% of cell death (IC_50_) was determined from respective dose–response curves.

### Statistical analysis

All experiments were obtained in triplicate, and the data was calculated as mean ± standard deviation (SD). Two-way ANOVA with Tukey’s test determined the significance of differences in means across groups, using the GraphPad Prism 9 software (San Diego, CA, USA). The *p* values < 0.05 were considered statistically significant.

## Results

### Characterization of Biosynthesized AgNPs

**Figure 1:**
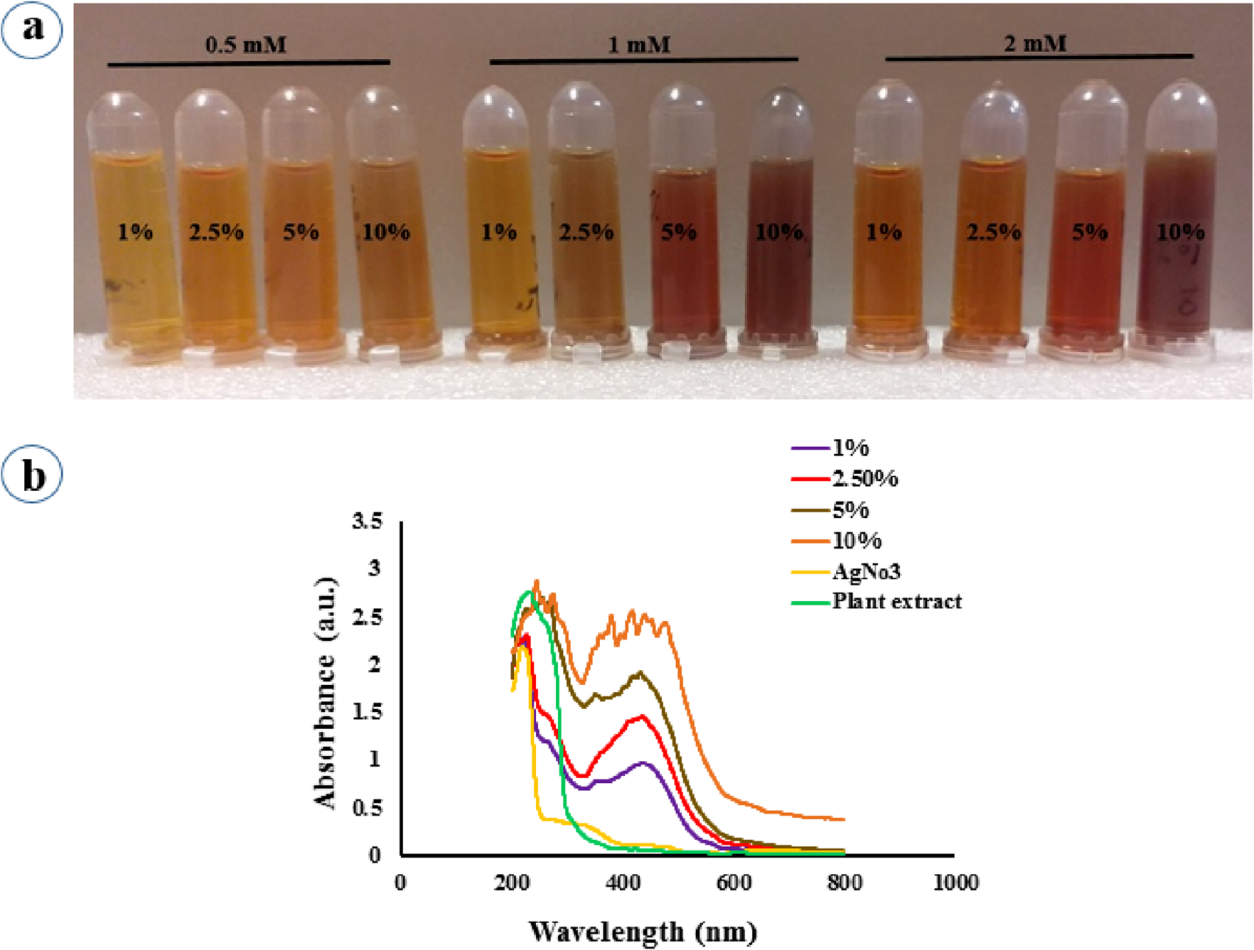
Characterization of Pf-AgNPs. (**a**) Visual observation of different color patterns and the reduction of Ag^+^ to Ag^0^ using the various concentration of aqueous leaf extract of *Perilla frutescens* and AgNO_3_ during 90 min. (**b**) UV–Visible spectroscopic analysis of AgNO_3_ (2 mM) alone and along with various concentration of the extract of *P. frutescens* (1, 2.5, 5, and 10%, v/v), and the extract of *P. frutescens* alone.

The bio-reduction process of Ag^+^ to Ag^0^ by aqueous leaf extract of *P. frutescens* as a reducing agent was shown through the color change of the reaction mixture from a pale yellow color to dim brown color during 90 min (Fig. 1a). As can be seen, the various concentrations of plant extract and AgNO_3_ affect the synthesis of Pf-AgNPs. Biosynthesis of Pf-AgNPs was confirmed via UV–Visible spectrophotometer with a maximum absorbance at 436 nm (Fig. 1b). This result is consistent with the plasmon resonance peak for the silver which has been reported in the range of 391–460 nm^20,39^. According to Fig. 1b, the most plant extract concentration (10%) exhibited maximum absorption along with several peaks causing more impurity in the production of NPs. In other words, the increase in absorption is associated with larger sizes and various shapes of NPs^40^. Therefore, the concentration of 2 mM AgNO_3_ and 2.5% plant extract were obtained as the optimum conditions.

### X-ray diffraction (XRD) analysis

**Figure 2:**
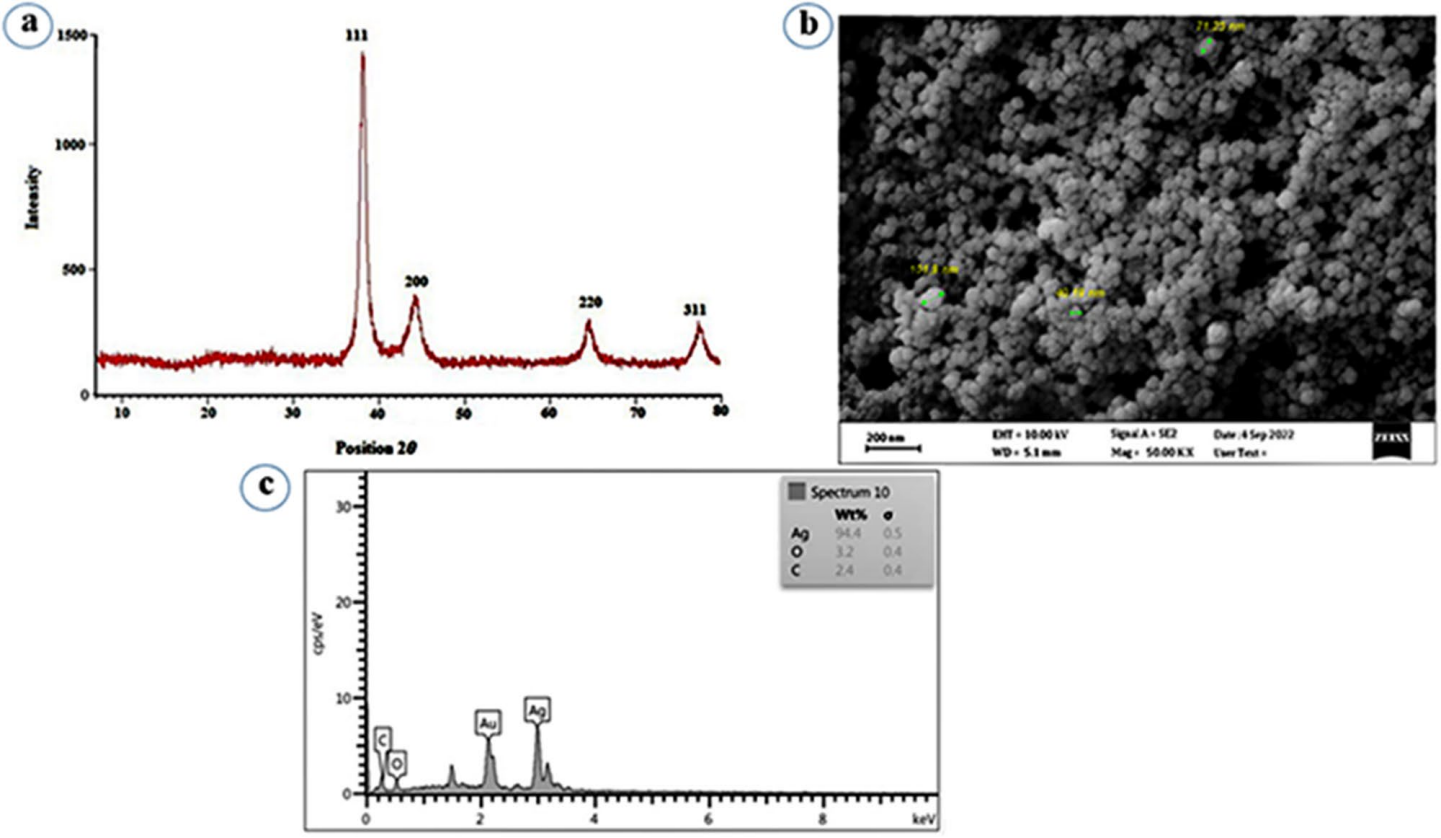
(**a**) XRD spectrum, (**b**) FESEM micrograph, (**c**) EDX spectrum of Pf-AgNPs.

The XRD pattern was used to identify the crystalline nature of Pf-AgNPs (Fig. 2). It has been shown four XRD peaks at 2*θ* values of 38.08° (111), 44.26° (200), 64.43° (220), and 77.35° (311) confirming the generation of the face centered cubic (FCC) structure of Pf-AgNPs (Fig. 2a)^21,41^. The crystallite size of Pf-AgNPs was estimated to be about 15 nm via Debye–Scherrer equation. Therefore XRD pattern proved pure and qualified synthesis of Pf-AgNPs. These results were in agreement with previous studies^18,19,42^.

### FESEM and EDX analysis

The surface morphology, size, and topography of Pf-AgNPs were observed by FESEM analysis coupled with EDX analysis. The FESEM images showed spherical and oval shaped Pf-AgNPs mostly with a diameter < 50 nm (Fig. 2b). As observed in Fig. 2b, the aggregation of some nanoparticles caused larger particles of Pf-AgNPs as a result of solvent evaporation during sample preparation and a natural tendency to create aggregates^42^. The analysis of EDX shown in Fig. 2c, indicated a strong signal of Ag peak at 3 keV that confirms the formation of Pf-AgNPs (94.4%). However, it was detected the presence of 3.2% of oxygen and 2.4% of carbon atoms in the Pf-AgNPs that prove chemical constituents of plant extract tend to bind on the surface of Ag ions as capping and stabilizing agents^43^.

### FTIR and Raman spectroscopy analysis

**Figure 3:**
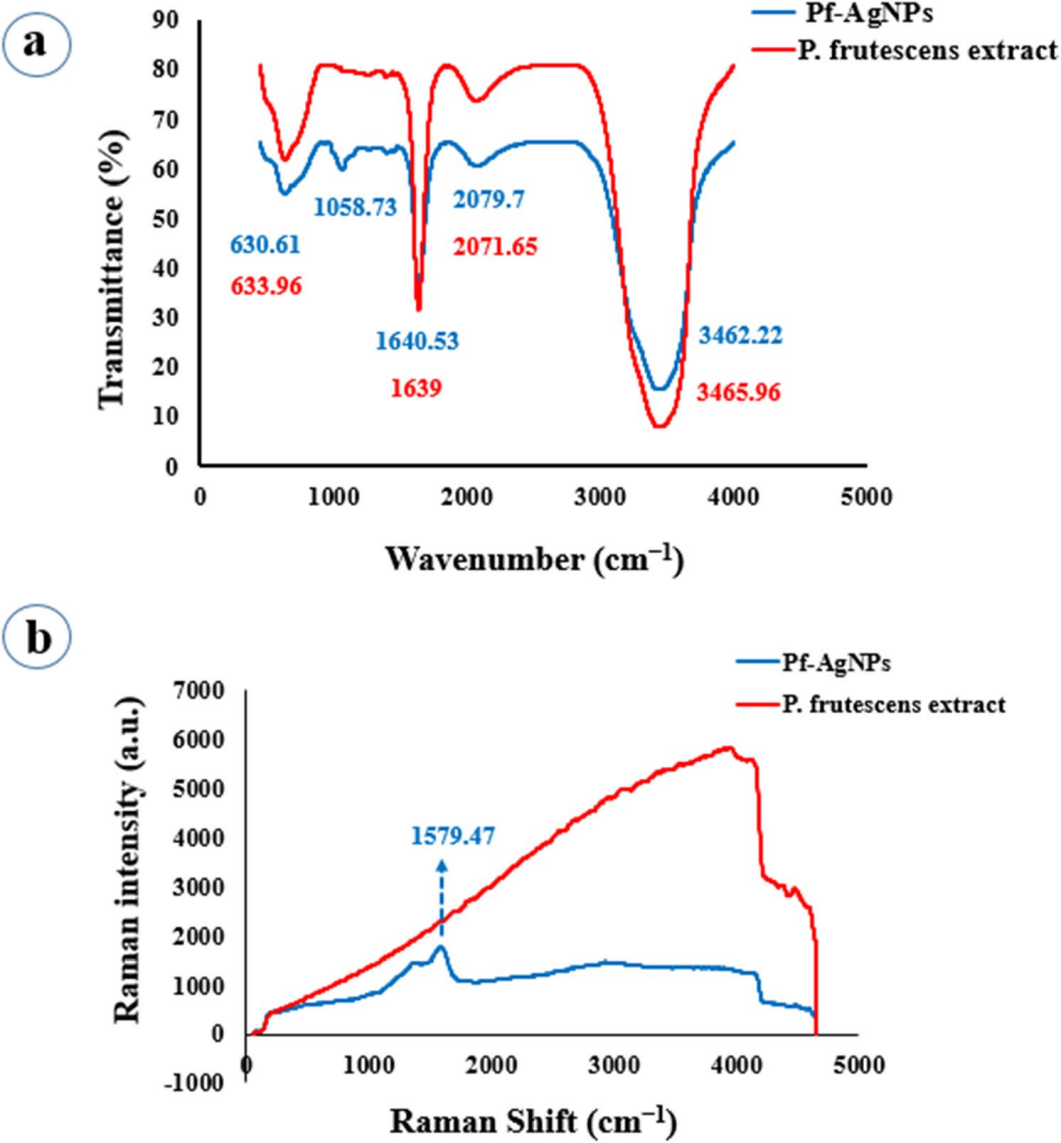
FTIR spectrum of the extract of *Perilla frutescens* and Pf-AgNPs (**a**), and Raman spectrum of the extract of *Perilla frutescens* and Pf-AgNPs (**b**).

The biomolecules responsible for reducing, capping, and stabilizing Pf-AgNPs were identified using the comparison of the FTIR spectra of *P. frutescens* extract and Pf-AgNPs (Fig. 3a). The FTIR spectra of Pf-AgNPs revealed several peaks around 3462.22, 2079.70, 1640.53, 1058.73, and 630.61 cm^−1^. The strong absorption peak at 3462.22 cm^–1^ is associated with the OH groups of phytochemicals of phenols and alcohols^44^. The peak at 2079.70 cm^–1^ represents the alkyne group and a peak at 1640.53 cm^−1^ can be allocated to amide C=O stretching^45^. A peak at 1058.73 cm^–1^ relevant to the amine group and C-O of alcohols^46^. According to de Araujo et al.^47^, the peak at 630.61 cm^−1^ represents the C-H groups of aromatic rings in the synthesis of Pf-AgNPs. Also, FTIR analysis of the *P. frutescens* extract showed four absorption peaks at 3465.96, 2071.65, 1639 and 633.96 cm^−1^. Therefore, there was a small extra peak (1058.73 cm^−1^) in the biosynthesized Pf-AgNPs that was not presented in the plant extract. Therefore, the main functional groups of hydroxyl, carbonyl, alkyne, amide, alcohols, and phenols were identified. Also, the shift of peaks confirmed the involvement of plant extract composition in the biosynthesis and stabilization of Pf-AgNPs^48^.

Raman spectroscopy is a useful technique to study the chemical structure of biomolecules contained in the plant extract and biomolecules added to the NPs solution by creating specific vibrational frequencies. Raman spectra of *P. frutescens* extract and Pf-AgNPs has been revealed in Fig. 3b which exhibit just a significant peak at 1579.47 cm^−1^ which is specific to AgNPs. It has been indicated that the spherical structure of AgNPs is a reason for creating unremarkable Raman peaks^20^. SERS spectroscopy has been rarely reported for biosynthesized AgNPs^19,49^. Indeed, the SERS experiments represented that the feature of (phyto)-functionalization of AgNPs permits other molecules to approach the NPs surface (a distance of a few nm) to “feel” the plasmon field of the NPs and this information is very important for other applications of the biosynthesized NPs^49^.

### Zeta potential and DLS

**Figure 4:**
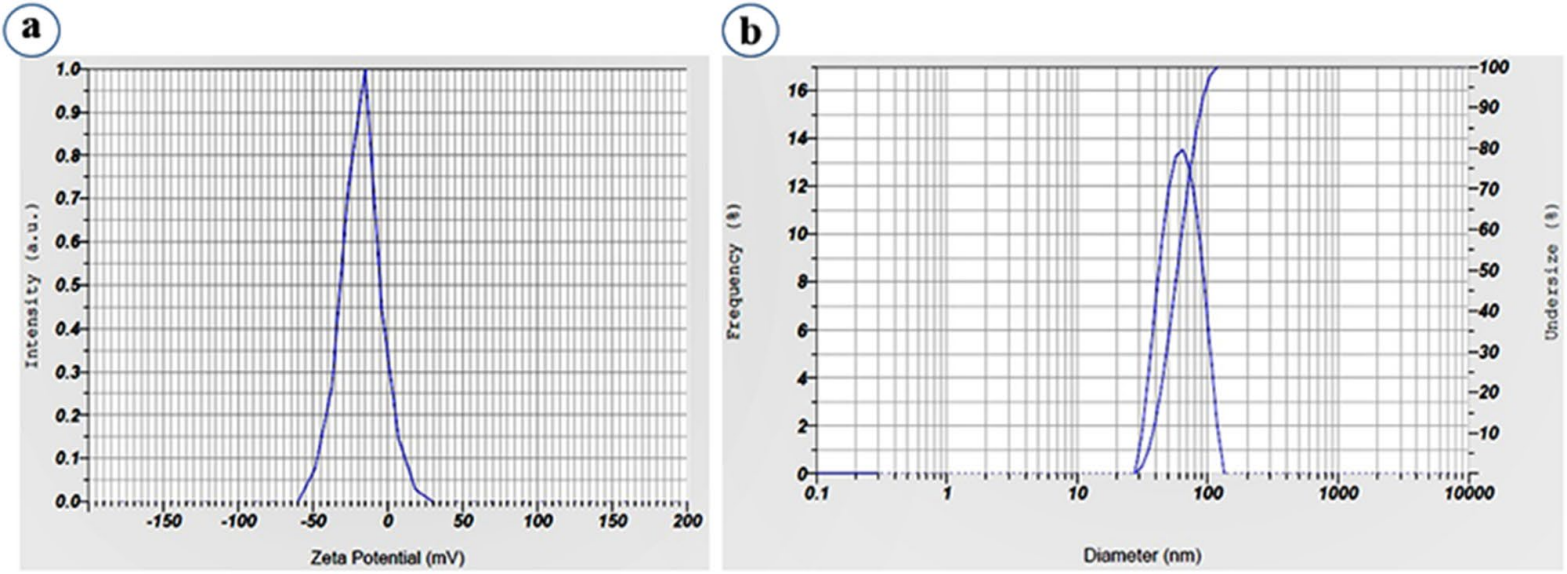
Zeta potential spectra measurements of biosynthesized Pf-AgNPs (**a**), and size distribution analysis of Pf-AgNPs (**b**).

Zeta potential (ζ) analysis was performed to evaluate electrostatic stability and surface potential of Pf-AgNPs in aqueous suspension (Fig. 4a). Zeta potential values of the biosynthesized Pf-AgNPs were evaluated to be − 18.1 ± 0.72 mV (Fig. 4a) indicating the particles moderate stability. Overall, nanoparticles with higher negative surface charge organize very stable colloids with very good dispersion without any mark of aggregation^50^.

The DLS is a technique for measuring the hydrodynamic diameter of nanoparticles and their agglomeration in the colloidal suspension. The average diameter of Pf-AgNPs was obtained from 28.3 to 161 nm and most Pf-AgNPs had a diameter under 61 nm with a Polydispersity Index (PI) value of 0.72 (Fig. 4b) that displays the possibility of aggregate. These results show a high dispersion in the size of the AgNPs that is attributable to the synthesis process. The results of DLS were in agreement with those of FESEM. However, the thickness of the hydration shell created in the aqueous ambiance of DLS analysis and the capping of phytochemicals over the NPs surface resulted in larger hydrodynamic diameter of Pf-AgNPs compared with FESEM and XRD analyses^20,51^.

### Phenolics content

**Figure 5:**
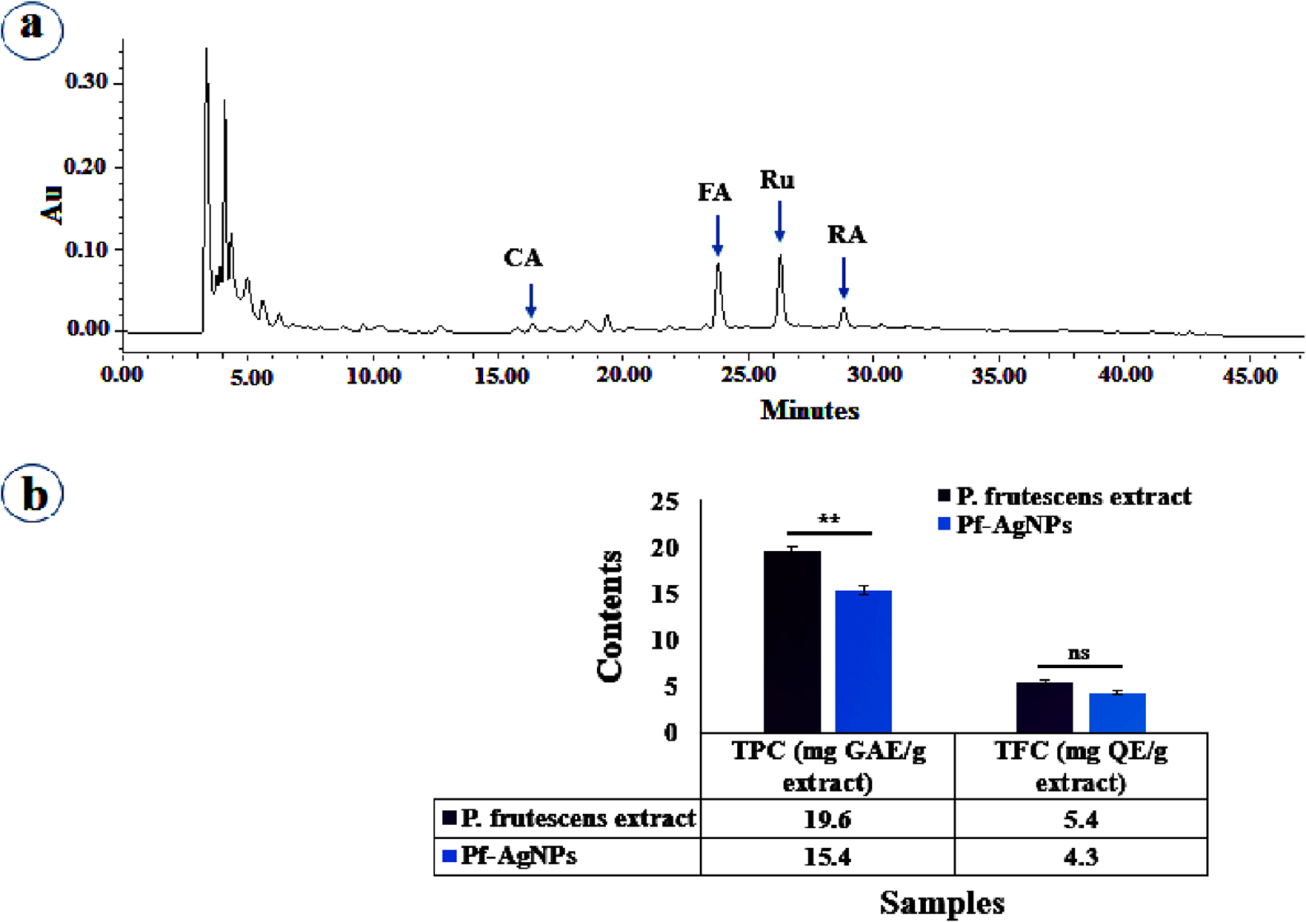
HPLC analysis of aqueous leaf extract of *Perilla frutescens* (**a**), and total phenol and flavonoid content of the extract of *Perilla frutescens* and Pf-AgNPs (**b**). *CA* caffeic acid, *FA* ferulic acid, *Ru* rutin, *RA* rosmarinic acid. Error bar represents standard deviation of mean. **p ≤ 0.01. Significant difference within a parameter between two lines is denoted by asterisk, and ns, means *p* > 0.05.

HPLC analysis of the aqueous leaf extract of *P. frutescens* confirmed the presence of the main phenolic compounds including ferulic acid (FA), rutin (Ru), caffeic acid (CA), and rosmarinic acid (RA) (Fig. 5a) that expect to be responsible for the bioreduction of Ag^+^ ions to Ag^0^. Although, there were many peaks in this chromatogram, four remarkable peaks related to CA, FA, Ru, and RA were identified through the comparison of their retention time with those of the available standards. (Fig. 5a). The contents of CA, FA, Ru, and RA obtained in amounts of 0.29 ± 0.01, 1.53 ± 0.07, 2.23 ± 0.10, and 0.96 ± 0.07 mg/g DW, respectively. Previous studies proved that phenolics content in leaf extract of *P. frutescens* is affected by extraction procedure and the type of solvent used^52,53^.

In this study, TPC (19.6 ± 0.50 mg GAE/g extract) and TFC (5.4 ± 0.20 mg QE/g extract) were higher in *P. frutescens* extract than Pf-AgNPs (Fig. 5b). The obtained results are consistent with other studies^19,54^. Polyphenol constituents as specialized metabolites of plants possess redox properties and many therapeutic beneficial effects that interact with AgNPS as reducing and capping agents^55^. Previous studies indicated the range of 86.45–450.83 µg of GAE/mg DE for TPC and 85.68–455.22 µg of RE/mg DE for TFC in leaf extract of *P. frutescens* based on the different solvents and its fractions^53^.

### Antioxidant activity using DPPH and FRAP assays

**Figure 6:**
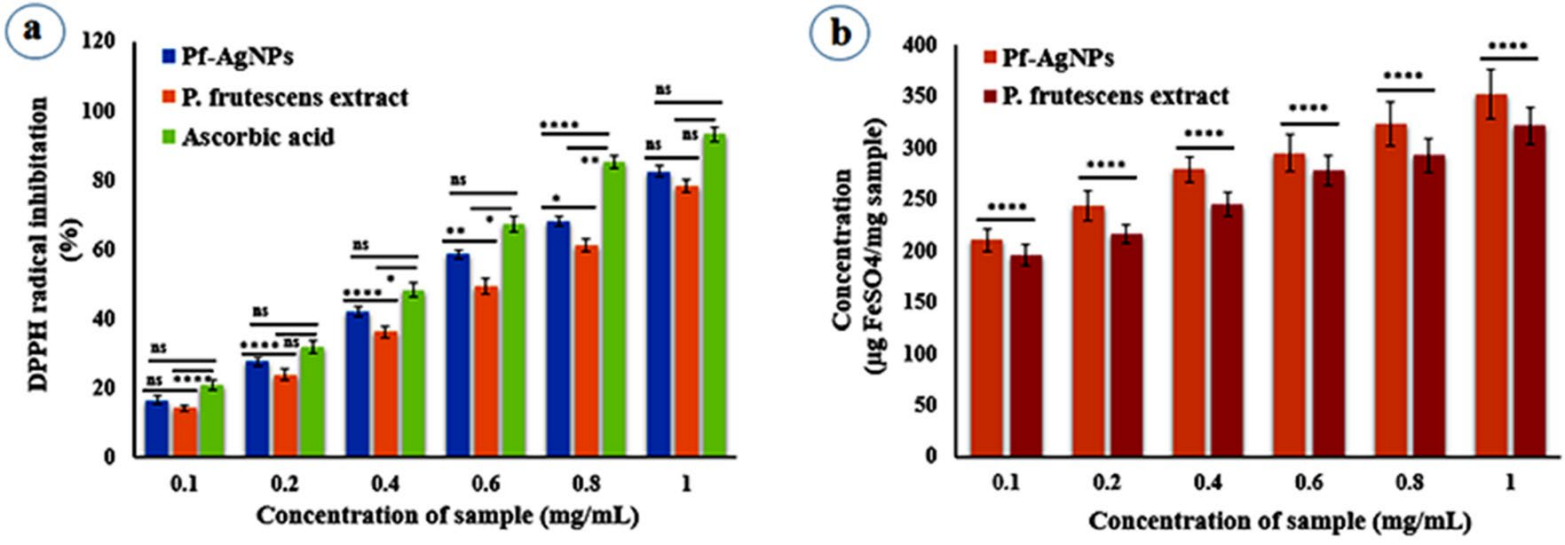
Antioxidant activity of the extract of *Perilla frutescens* and Pf-AgNPs (**a**) DPPH, (**b**) FRAP. Error bar represents standard deviation of mean. *p ≤ 0.05, **p ≤ 0.01, ****p ≤ 0.0001. Significant difference within a parameter between two lines is denoted by asterisk, and ns, means *p* > 0.05.

Natural antioxidants including phenolics, terpenoids, and alkaloids possess an extraordinary desirable position in improving human health by reducing or stopping various diseases through the detoxification of oxygen free radicals, lowering genetic mutations, and averting cell damage^56^. In this study, the antioxidant activity of Pf-AgNPs and leaf extract of *P. frutescens* was evaluated by DPPH and FRAP assays. The DPPH scavenging activity by *P. frutescens* extract, Pf-AgNP, and ascorbic acid as the standard were determined at six different concentrations (0.1–1 mg/mL) ranging from 14.10 to 78.44%, 16.44 to 82.54%, and 20.76 to 93.27%, respectively (Fig. 6a). The DPPH scavenging activity was determined according to the change in color and it enhanced in a dose dependent manner. The DPPH radical inhibition of Pf-AgNPs was higher than *P. frutescens* extract and comparable with the ascorbic acid standard, and the IC_50_ values of 0.52 ± 0.06, 0.62 ± 0.09, and 0.45 ± 0.03 mg/mL, respectively, were obtained.

In addition, FRAP assay indicated the concentration dependent activity of Pf-AgNP and *P. frutescens* extract at six different concentrations (0.1–1 mg/mL) ranging from 210.43 to 352.12 and 195.97 to 321.45 µg FeSO4/mg sample, respectively (Fig. 6b). The results indicated that Pf-AgNPs possess higher reducing activity (the reduction of TPTZ-Fe (III) to TPTZ-Fe (II) complex) than *P. frutescens* extract (Fig. 6b). The remarkable antioxidant activity of Pf-AgNPs with both FRAP and DPPH assays may be ascribed to the presence of varied phytochemicals, specially phenolic acids and flavonoids. These phytochemicals as a capping agent can get adsorbed onto AgNPs surfaces and thereby interacts and scavenge free radicals effectively^57^.

### Antimicrobial activity

It has been reported broad applications from antimicrobial properties of AgNPs including environmental usage, the health industry, food storage, textile coatings, and antiseptic creams^58^. In the present study, the antimicrobial activity of *P. frutescens* extract and Pf-AgNPs was evaluated using the microdilution method on the strain of a Gram-negative bacterium (*E. coli*), the strain of a Gram-positive bacterium (*S. aureus*), and a pathogenic fungus (*C. albicans*). The values of MIC, MBC, and MFC were obtained for both *P. frutescens* extract and Pf-AgNPs (Table 1). The results indicated that Pf-AgNPs had significantly the higher antibacterial activity (MIC of 0.78 mg/mL) against both bacterial strains compared to *P. frutescens* extract (MIC of 6.25 mg/mL). Nevertheless, higher bactericidal activity of Pf-AgNPs against *E. coli* (MBC of 1.56 mg/mL) than *S. aureus* (MBC of 6.25 mg/mL) was observed while *P. frutescens* extract indicated no bactericidal activity up to 12.5 mg/mL. Also, the fungus *C. albicans* was more sensitive to Pf-AgNPs with MIC of 8 mg/mL and MFC of 16 mg/mL compared to *P. frutescens* extract with MIC of 32 mg/mL and without fungicidal activity.

**Table 1:** Antimicrobial activity of *Perilla frutescens* (L.) extract and Pf-AgNPs (mg/mL).

Samples	*E. coli*		*S. aureus*		*C. albicans*	
	MIC	MBC	MIC	MBC	MIC	MFC
*P. frutescens* extract	6.25	NA	6.25	NA	32	NA
Pf-AgNPs	0.78	1.56	0.78	6.25	8	16
Safexim^a^	0.5	1	2	4	–	–
Nystatin^a^	–	–	–	–	32	64

*NA* not active.

^a^MIC and MBC of antibiotics were presented as μg/mL.

### Biocompatibility and cytotoxicity analysis

**Figure 7:**
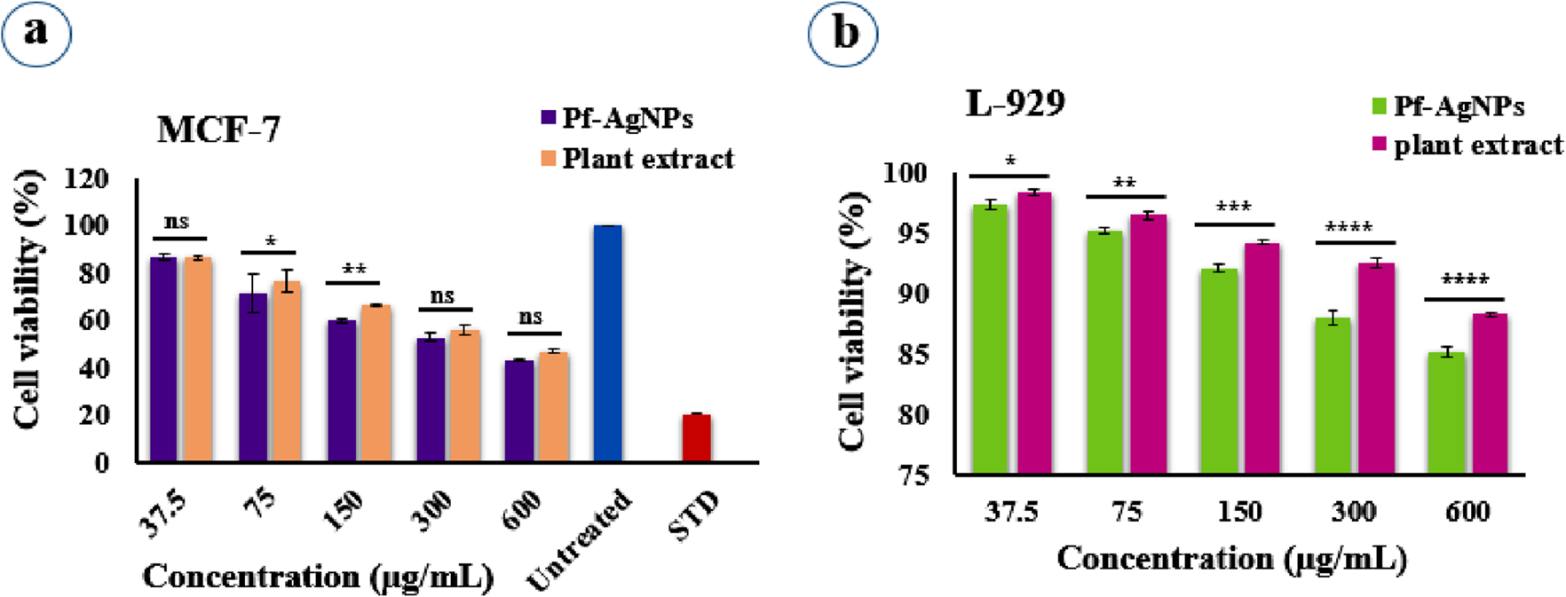
Cell viability of MCF-7 (**a**) and L-929 (**b**) after treatment with different concentrations of Pf-AgNPs and the extract of *Perilla frutescens*. Error bar represents standard deviation of mean. *p ≤ 0.05, **p ≤ 0.01, ***p ≤ 0.001, ****p ≤ 0.0001. Significant difference within a parameter between two lines is denoted by asterisk, and ns, means *p* > 0.05.

**Figure 8:**
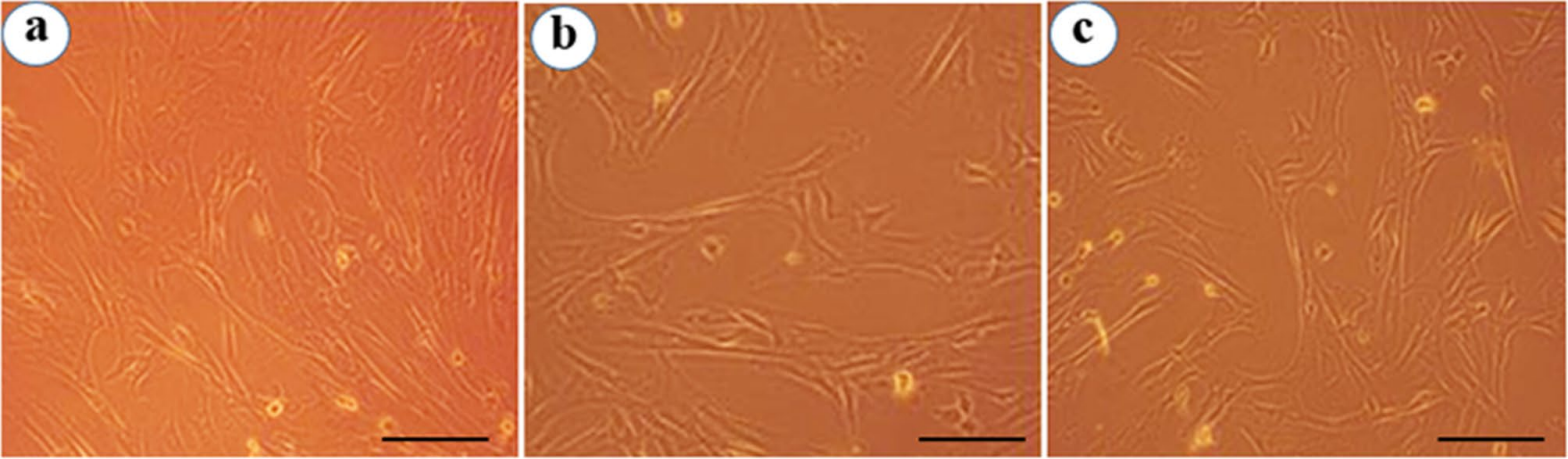
Cytotoxic activity of the highest concentration Pf-AgNPs and the extract of *Perilla frutescens* (600 µg/mL) on L-929 cells. (**a**) Untreated cells, (**b**) Pf-AgNPs, (**c**) the extract of *P. frutescens*. (Scale bars represent 200 µm).

The dose-dependent response of Pf-AgNPs and *P. frutescens* extract with various concentrations (37.5–600 µg/mL) on normal fibroblast cells (L-929) viability was evaluated using an MTT assay for 24 h (Fig. 7b). The results revealed that Pf-AgNPs and *P. frutescens* extract at the highest concentration of 600 µg/mL decreased the viability of the L-929 cells to 85.10 and 88.20%, respectively (Fig. 8). Therefore, results reveal that Pf-AgNPs and plant extract are relatively nontoxic to normal cells indicating the biocompatibility of them with L-929 cells.

**Figure 9:**
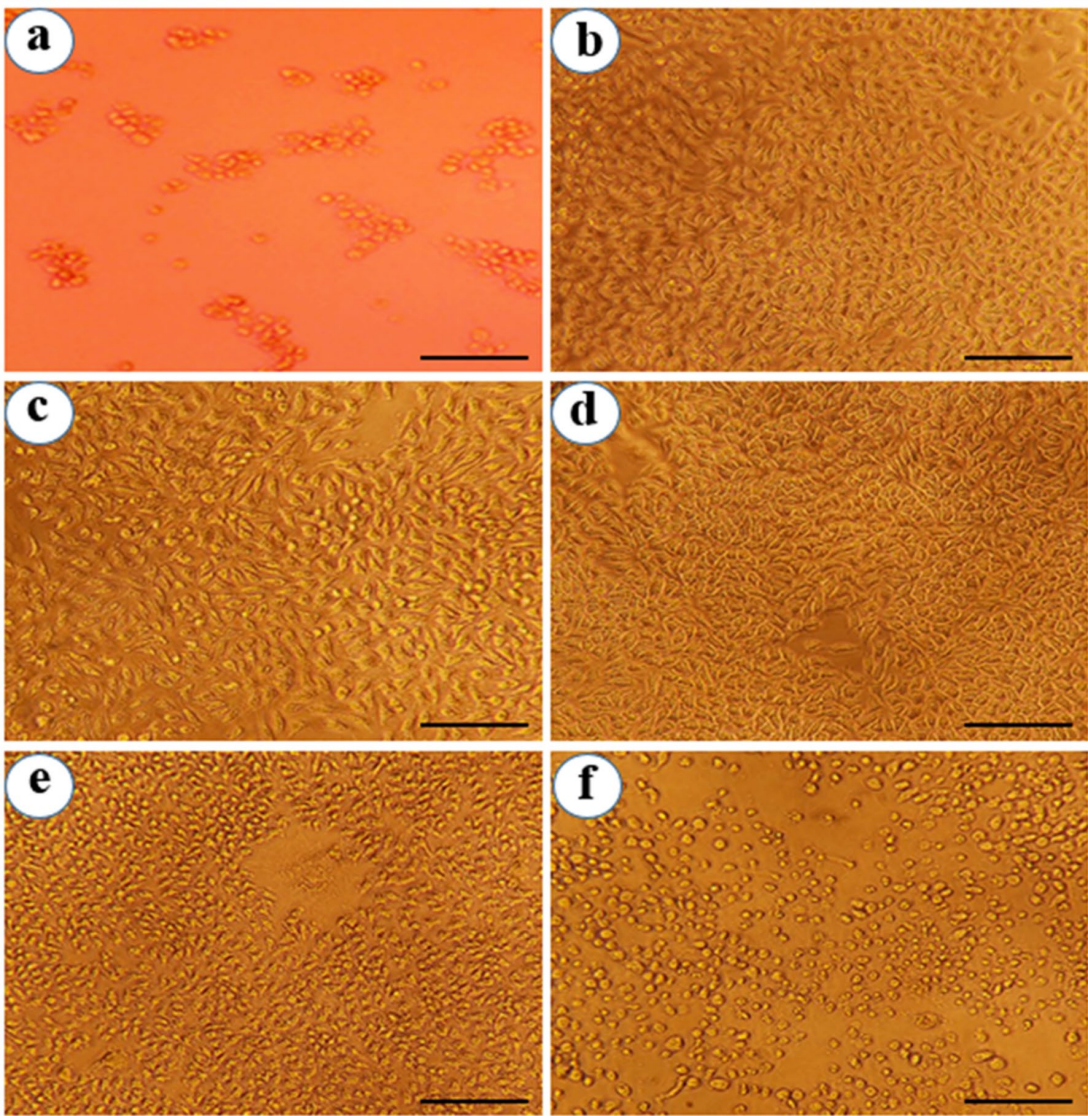
Cytotoxic activity of the different concentrations of Pf-AgNPs on MCF-7 cells. (**a**) Standard (75 µg/mL), (**b**) 37.5 µg/mL, (**c**) 75 µg/mL, (**d**) 150 µg/mL, (**e**) 300 µg/mL, (**f**) 600 µg/mL of Pf-AgNPs. (Scale bars represent 200 µm).

**Figure 10:**
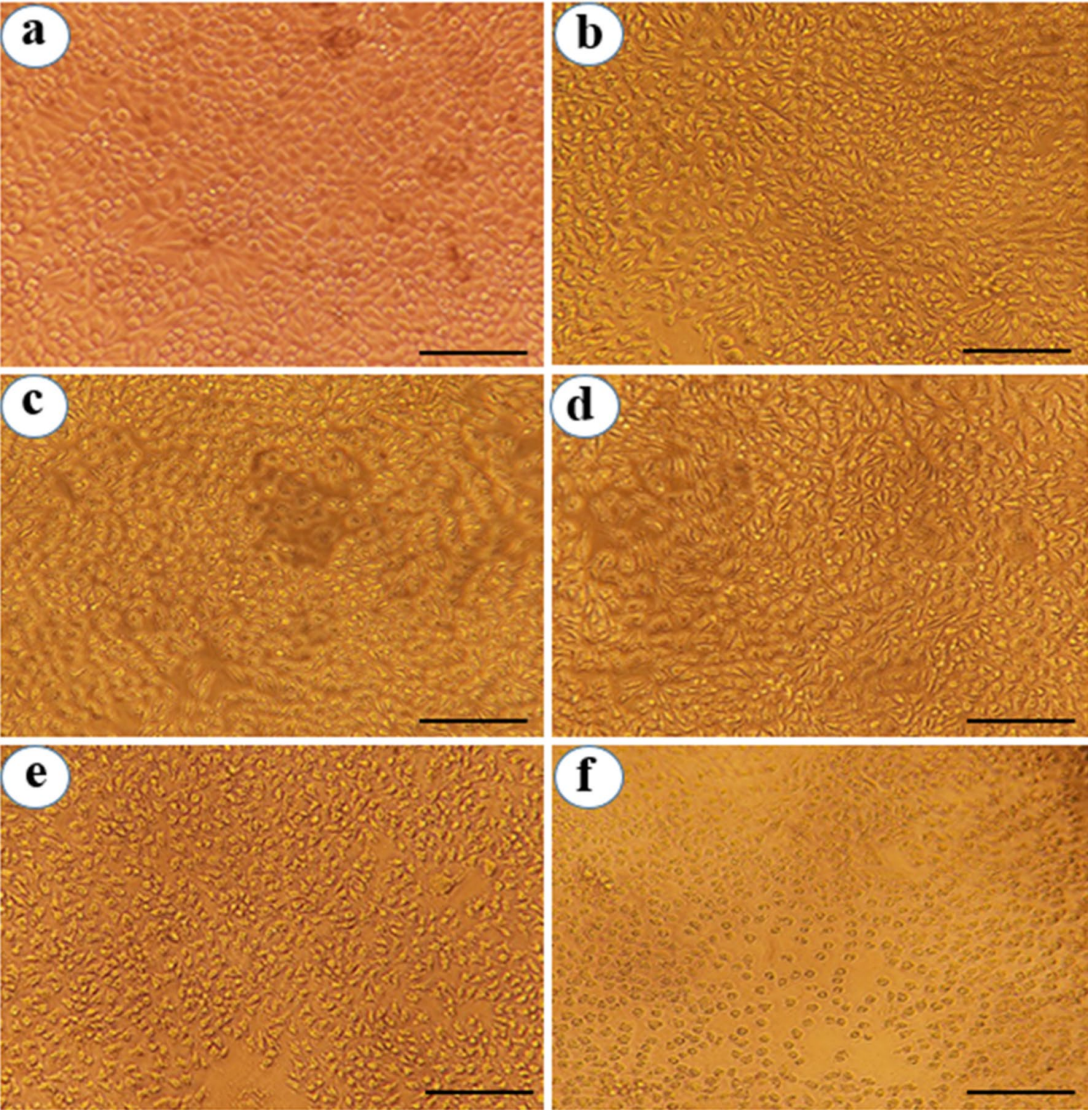
Cytotoxic activity of the different concentrations of the extract of *Perilla frutescens* on MCF-7 cells. (**a**) Untreated cells, (**b**) 37.5 µg/mL, (**c**) 75 µg/mL, (**d**) 150 µg/mL, (**e**) 300 µg/mL, (**f**) 600 µg/mL of the extract of *P. frutescens*. (Scale bars represent 200 µm).

In other hand, cytotoxic activity of *P. frutescens* extract and Pf-AgNPs was evaluated against MCF-7 breast cancer cells with various concentrations (37.5–600 µg/mL) using an MTT assay for 24 h (Fig. 7a). It was revealed that Pf-AgNPs (Fig. 9) and *P. frutescens* extract (Fig. 10) at the highest concentration of 600 µg/mL reduced the viability of the MCF-7 cells to 43.15 and 46.75%, respectively which indicated their moderate toxicity on MCF-7 cells. Although, the significant difference (p < 0.05) was not observed within the percentage of viable cancer cells between Pf-AgNPs and *P. frutescens* extract, the IC_50_ value of Pf-AgNPs (346.2 µg/mL) against MCF-7 cells was lower than *P. frutescens* extract (467.4 µg/mL) (Fig. 8). Therefore, both Pf-AgNPs and plant extract may be had a potential for inhibiting MCF-7 cell proliferation. The drug cisplatin standard decreased the viability of the MCF-7 cells to 20.36% at the concentration of 75 µg/mL and at 24 h of incubation period (Fig. 9).

## Discussion

In the present study, the aqueous leaf extract of *P. frutescens* (5% dilution) as a reducing agent and 2 mM AgNO_3_ with a 1:40 ratio, 60 °C temperature, and 90 min time were considered as the optimum conditions for the biosynthesis of Pf-AgNPs. In general, factors such as the quiddity of plant extract and its concentration, metal salt concentration, contact duration, temperature, and pH are well recognized to affect the quantity and rate of synthesis of NPs and their other characteristics^59^. Behravan et al.^60^ studied different concentrations of AgNO_3_ (0.5, 1, 3, and 10 mM) and plant extract (3, 5, 10, 15, and 30 mL) in several contact times (1, 2, 6, 12 and 24 h). They indicated that AgNPs with spherical shape and a size of 30 to 70 nm were obtained at 3 mM AgNO_3_, 5 mL of aqueous extract, and a time of 1 h as optimum conditions. Also, Mohseni et al.^61^ reported that different volume ratios of AgNO_3_ (1 mM) salt to pomegranate seed extract (1:1, 1:9, 1:4, and 3:7 ratios) had different absorptions and the ratio of 1:9 showed the highest and the most appropriate absorption and thereby AgNPs were formed with the size range of 19.97 to 54.48 nm. The effect of pH on the AgNPs synthesis in various plants is different, however, it has been reported that AgNPs with the suitable size and shape, and also low assembly occur at pH values ranging from neutral to basic^62^. In addition, the time and temperature of the reaction influence NPs synthesis. By reducing reaction time and increasing temperature, the NPs with smaller sizes were obtained^63^.

The synthesis of Pf-AgNPs was confirmed by UV–Visible spectroscopy, XRD, FESEM, EDX, zeta potential, DLS, SERS, and FTIR analysis. The synthesized Pf-AgNPs had the optimal size (< 61 nm), shape (spherical), and moderate stability (− 18.1 mV). Accordingly, the synthesis and stability of NPs along with various morphologies affect by parameters including the nature and concentration of phytochemicals, metal salt concentration, temperature, pH, and time^64^. The change of each one of these parameters alters the shape, size, and morphology of NPs. Therefore, the precise optimization of the NPs synthesis process is necessary to obtain the appropriate shape, size, and morphology of NPs.

The hydroxyl and carbonyl groups of phenolic compounds interact with silver ions through chelation and they are responsible for stabilizing the NPs^62^. The stabilization of NPs inhibits their further agglomeration^3^. The mechanism of metallic NPs synthesis such as AgNPs through plant extracts could categorize into three steps: (1) the reduction (bioreduction of Ag^+^ to Ag^0^ by compounds of phenolics, terpenoids, proteins, enzymes, cofactors, etc.), (2) the growth (the aggregation of Ag atoms), (3) the stabilization (capping of NPs surface using bioactive compounds)^65^. In this study, HPLC and spectroscopic analysis of plant extract of *P. frutescens* confirmed that phenolic compounds are responsible for the synthesis and stabilization of AgNPs. Polyphenolic compounds identified in *P. frutescens* extract such as rosmarinic acid, caffeic acid, ferulic acid, and rutin cause the gathering of silver ions into smaller Pf-AgNPs. Indeed, oxidation of phenolic compounds using AgNO_3_ creates stable phenoxy radicals and decreases metallic silver atoms that these atoms join together and form a stable silver nanoparticle^27^. In addition, an interesting consequence was that although the TPC and TFC of Pf-AgNPs were lower than those of plant extract, the antioxidant activity of Pf-AgNPs was higher compared to plant extract. It is probably due to the unique chemical, physical and biological properties of AgNPs.

Furthermore, Pf-AgNPs had high antimicrobial activity against *E. coli* and *S. aureus* (MIC = 0.78 mg/mL), and *C. albicans* (MIC = 8 mg/mL) while the plant extract showed low antimicrobial activity against both bacterial strains and the fungus tested. The antimicrobial activity of Pf-AgNPs can be affected by the shape, size, zeta potential, concentration, and colloidal state of AgNPs, as well as conditions of their synthesis including ions, pH, and macromolecules^44,66^. Generally, two main parameters of NPs, a reduced size and a positive zeta potential, cause the highest antimicrobial activity. Indeed, NPs with a smaller diameter (< 30 nm) and a more positive zeta potential will have a higher interaction and permeability to the bacteria membrane with a negative surface charge, thereby they will have a higher antimicrobial activity^67^. In this study, Gram-negative bacteria were more sensitive to Pf-AgNPs than Gram-positive bacteria. Indeed, Gram-positive bacteria possess a thicker peptidoglycan layer compared to Gram-negative bacteria which lead to difficult penetration of AgNPs into Gram-positive bacteria^68^. Behravan et al.^60^ reported that AgNPs synthesized with *Berberis vulgaris* extracts had a very higher antibacterial effect on *E. coli* bacteria than *S. aureus* bacteria. However, Pirtarighat et al.^9^ revealed that Gram-negative bacteria against AgNPs are more resistant than Gram-positive bacteria. Although, it is expected that the leaf extract of *P. frutescens* indicated a significant antimicrobial activity, it showed low activity. That is probably due to the type of used solvent (water) and the extraction procedure (heat up)^45^.

In this study, Pf-AgNPs and plant extract are relatively nontoxic to normal cells indicating the biocompatibility of them with L-929 cells. Also, the IC_50_ value of Pf-AgNPs (346.2 µg/mL) against MCF-7 cells was lower than *P. frutescens* extract (467.4 µg/mL) (Fig. 7a,b). Similarly, Mohanta et al.^69^ reported that the IC_50_ values for biosynthesized GE-AgNP treatment were significantly lower in MDA-MB-231 cells than in L-929 cells after exposure for 24 or 48 h. Also, the biosynthesized AgNPs using *Pyrostegia venusta* leaf aqueous indicated significantly increased cytotoxic activity against COS-7 cells in vitro, with IC_50_ value of 50.48 µg/mL^70^. Heidari et al.^71^ reported that *Thymus vulgaris* L. extract possesses less toxicity (70% cell mortality) on T47D breast cancer cells than biosynthesized AgNPs (90% cell mortality). In addition, it has been reported that green synthesized AgNPs (20–52 nm) from the extract of *Tamarindus indica* L. indicated 50% cell death of MCF-7 at 20 μg/mL^38^. Therefore, it can be inferred that the effect of biosynthesized AgNPs on cancer cells can be affected by extract compounds used in their synthesis and also their size and shape. In addition, it has been reported that AgNPs may damage cell integrity by disrupting the mitochondrial respiratory chain, increasing reactive oxygen species (ROS) production, and ATP synthesis, thereby resulting in the death of cancer cells^71^.

## Conclusions

Plant-mediated biosynthesis of NPs has been remarkably noticed as a low-cost and eco-friendly source. In the present study, efficient production of AgNPs was obtained using the green reducing and capping biomolecules from aqueous leaf extract of *P. frutescens*. The biosynthesis of Pf-AgNPs was confirmed using different analysis methods including UV–Vis spectroscopy, FESEM, XRD, EDX, Zeta potential, DLS, Raman, and FTIR. The leaf extract of *P. frutescens* and Pf-AgNPs indicated remarkable antioxidant activity in both DPPH and FRAP assays. Besides this Pf-AgNPs demonstrated high antibacterial properties against *E. coli* and *S. aureus* (MIC of 0.78 mg/mL) and good antifungal activity against *C. albicans* (MIC of 8 mg/mL). Also, Pf-AgNPs and *P. frutescens* extract indicated moderate toxicity on MCF-7 breast cancer cells that IC_50_ value of Pf-AgNPs (346.2 μg/mL) against MCF7 cells was lower than *P. frutescens* extract (467.4 µg/mL). Overall, the phytochemicals of *P. frutescens* extract were not only efficient in reducing and stabilizing Pf-AgNPs but also played a main role in the antimicrobial, antioxidant, and anticancer activities of Pf-AgNPs. Therefore, biosynthesized Pf-AgNPs could have potential biomedical and pharmaceutical applications with no or minimal side effects and also without damaging the environment in the future.

## Supplementary Information

Supplementary Information.

## Data Availability

All data generated or analysed during this study are included in this published article [and its Supplementary Information files].
